# A Major Role for the Lateral Habenula in Depressive Illness: Physiologic and Molecular Mechanisms

**DOI:** 10.3389/fpsyt.2019.00320

**Published:** 2019-05-22

**Authors:** Philip W. Gold, Bashkim Kadriu

**Affiliations:** National Institute of Mental Health (NIMH), National Institute of Health, Bethesda, MD, United States

**Keywords:** ketamine, major depressive disorder, lateral habenula, HPA axis, NMDA-type receptors, sleep

## Abstract

Emerging preclinical and clinical evidence indicate that the lateral habenula plays a major role in the pathophysiology of depressive illness. Aberrant increases in neuronal activity in the lateral habenula, an anti-reward center, signals down-regulation of brainstem dopaminergic and serotonergic firing, leading to anhedonia, helplessness, excessive focus on negative experiences, and, hence, depressive symptomatology. The lateral habenula has distinctive regulatory adaptive role to stress regulation in part due to its bidirectional connectivity with the hypothalamic–pituitary–adrenal (HPA) axis. In addition, studies show that increased lateral habenula activity affects components of sleep regulation including slow wave activity and rapid eye movement (REM), both disrupted in depressive illness. Lack of perceived reward experienced during the adverse outcomes also precipitates lateral habenula firing, while outcomes that meet or exceed expectations decrease lateral habenula firing and, in turn, increase midbrain dopaminergic and serotonergic neurotransmission. The ability to update expectations of the environment based on rewards and aversive stimuli reflects a potentially important survival mechanism relevant to the capacity to adapt to changing circumstances. What if one lives in a continuously aversive and invalidating environment or under the conditions of chronic stress? If there is a propensity of the habenula to release many burst discharges over time, an individual could habitually come to perceive the world as perpetually disappointing. Conceivably, the lateral habenula could learn to expect an adverse outcome systematically and communicate it more easily. Thus, if the lateral habenula fires more frequently, it may lead to a state of continuous disappointment and hopelessness, akin to depression. Furthermore, postmortem studies reveal that the size of the lateral habenula and total number of neurons are decreased in patients who had depressive illness. Novel research in the field shows that ketamine induces rapid and sustained antidepressant effect. Intriguingly, recent preclinical animal models show that ketamine abolishes *N*-methyl-*D*-aspartate receptor (NMDAR)-dependent lateral habenula bursting activity, leading to rapid resolution of depressive symptoms.

## Introduction

The habenula is a component of the diencephalon and, together with the pineal gland, makes up the epithalamus (1). This evolutionary well-conserved structure plays a central role connecting forebrain and midbrain (2). It is involved in multiple processes that are core components of the major depressive syndrome such as reward processing, cognition, stress adaptation, sleep and circardian rythm regulation, biological rhythms, and the regulation of monoaminergic (dopamine and serotonin) neurotransmission (2–6). Its dysfunction has been implicated in psychiatric illnesses closely related to maladaptive processing of positive and negative valence (7). Further, lateral habenula serves as an interface among emotions, stressors, and cognitions. The hypothalamic–pituitary–adrenal (HPA) axis and lateral habenula appears to have unique bidirectional regulation, thus perturbations of the HPA axis are associated with alterations of lateral habenula function (5).

### Lateral Habenula and Reward Processing in Depression: Interaction With Dopaminergic and Serotoninergic Neurotransmission

One of the functions of the lateral habenula is to encode negative motivational values associated with primary punishment in humans (8) and primates (9, 10). Thus, the habenula encodes the values of cues previously paired with an aversive outcome. Accordingly, habenula responses predict the extent to which individuals withdraw or approach negative and positive cues, respectively, thus playing a central role in driving aversive motivated learning and behavior (7, 11). This is accomplished through the extensive connections of the lateral habenula neurons (largely glutamatergic) to dopamine neurons in the ventral tegmental area (VTA) and the substantia nigra (8, 9). In addition, the rostromedial tegmental nucleus (RMTg) also known as the GABAergic tail of the VTA, receives glutamatergic inputs from the lateral habenula and sends substantial GABAergic projections to the midbrain dopaminergic system including VTA, as well dorsal raphe nucleus, locus coeruleus and other regions. Notably, VTA receives direct projections from the lateral habenula as well (6, 7, 9).

When a reward is smaller than expected or the anticipated reward is unsatisfactory, the firing rate of the lateral habenula increases, leading to inhibition of dopamine release from midbrain dopaminergic neurons that project to the nucleus accumbens, highly involved in reward processing (2, 7, 12, 13). Therefore, the lateral habenula is implicated in encoding information about aversive signals or missing rewards. Its firing rate increases in response to chronic stress, punishment, and stimuli that have been previously associated with negatively charged experiences (7, 11). Accordingly, the lateral habenula plays a key function in learning from painful experiences and in making decisions to avoid such aversive experiences in the future (12, 13). In contrast, if the expected reward meets or exceeds our expectations, the firing rate of the lateral habenula decreases, leading to activation of brainstem dopaminergic nuclei, which activate the nucleus accumbens (2, 12–14) that is critically important for mediating and experiencing reward. This activity is thought to help us remember the details of how we obtained the reward. Thus, this will also help us to remember how to get the reward in the future (7). When the encoding of the reward becomes hyperactive, it can result in obsessive reward-seeking behaviors involved in addiction disorders (15).

Lateral habenula also interacts with the raphe nuclei and the serotonergic system. When the firing rate of the lateral habenula is high, the release of serotonin from the raphe nuclei is reduced, resulting in decreased serotonin neurotransmission. Input to the lateral habenula from the basal ganglia increases the firing rate of the lateral habenula, leading to aversive outcomes, but this pathway is suppressed by serotonin (16, 17).

The ability to update expectations of the environment based on rewards and aversive stimuli reflects a potentially important survival mechanism relevant to the capacity to adapt to changing circumstances (12, 13). Theoretically, if one lives in a continuously highly aversive and invalidating environment or under the conditions of chronic stress, there will be a propensity of the habenula to release many burst discharges over time, so that an individual could systematically come to perceive the world as perpetually disappointing (12, 13, 18). Conceivably, the habenula could learn to expect an adverse outcome systematically and communicate it more easily. Thus, Kaye et al. note that if the habenula fires more frequently, it may lead to a state of continuous disappointment and hopelessness, akin to depression. In addition, lateral habenula neuronal activity is significantly enhanced in rodent animal models of depression (13, 19) as well as in depressed patients (20–22).

Given its unique capability to relay information from limbic forebrain to midbrain monoamine nuclei *via* high-density afferents to monoaminergic centers, the lateral habenula could potentially induce the down-regulation of the serotonergic, noradrenergic, and dopaminergic systems. This complex process, resulting from functional hyperactivation of the lateral habenula, has critical implications for regulating aversive behaviors and depressive pathophysiology (2, 16, 23, 24).

### Bidirectional Relationship Between Activation of the Hypothalamic–Pituitary–Adrenal Axis and the Lateral Habenula

The paraventricular nucleus of the hypothalamus contains abundant corticotropin-releasing hormone (CRH) neurons, which release CRH to activate the pituitary–adrenal axis. The paraventricular nucleus of the hypothalamus sends direct projections to the lateral habenula, but the functional consequences of this projection are unknown (3, 23). During increased stress and adverse experiences, the HPA axis and the lateral habenula are concomitantly activated (25), but it is not clear, however, which occurs first.

Direct activation of the lateral habenula is associated with HPA axis activation (25, 26). As a corollary, bilateral lesioning of the lateral habenula abolishes the HPA axis and behavioral responses to stress (27). The extent to which these phenomena reflect direct interactions between the lateral habenula and the hypothalamic CRH neurons or proceed by intermediary pathways ultimately linking the habenula to hypothalamic CRH neurons and *vice versa* is currently unknown.

The lateral habenula also expresses CRH receptor 1 (CRHR1) receptors, which are activated *via* restraint stress. CRH activates the lateral habenula (23). Although this stress-mediated activation of habenula CRH receptors is likely to participate in the stress response, this premise remains to be validated. To this end, work from Authement et al., show that CRH-mediated physiological stimulation in slices or behavioral maternal deprivation in rodent pups decreased the abundance of potassium channels in pups, which in turn increases the firing rate of lateral habenula neurons (23). Further work is required to elucidate the relationship among CNS pathways that mediate the relationship between hypothalamic and extrahypothalamic CRH neurons and their consequent behavioral and physiological effects.

### Impact of Lateral Habenula on Sleep Alterations in Depression

Sleep is another domain that is systematically disrupted in patients with depressive illness. Most notably, patients with depressive illness have decreased slow wave sleep activity and a systematic increase in rapid eye movement (REM) sleep, as well as a faster onset of REM sleep than controls (28). Specifically, the lateral habenula appears to have a critical role in regulating oscillatory theta hippocampal activity through modulation of temporal dopaminergic and serotoninergic firing pattern, suggesting a synaptic mechanism for memory consolidation during REM sleep, a decrease in slow wave sleep, and a highly significant increase in REM sleep (16, 28). Thus, an increase in the activity of the lateral habenula would result in the decrease in slow wave sleep and the significant augmentation of REM sleep, well-known characteristics of depressive illness.

#### Glutamatergic Modulation and the Lateral Habenula: Evidence From Preclinical and Clinical Studies

One of the most exciting paradigm shifts in biological psychiatry in the past two decades is the discovery that a single subanesthetic dose of ketamine (a prototypic glutamatergic modulator) to treatment-resistant depressed patients induces rapid and sustained antidepressant responses within hours, often lasting as long as 1–2 weeks (29). This initial hypothesis surrounding the fast-onset antidepressant response was related to direct and indirect *N*-methyl-D-aspartate receptor (NMDAR) inhibition in the hippocampus and medial prefrontal cortex, which in turn induced a rapid increase in neuroplasticity and neurogenesis (30, 31). However, recent animal work regarding the mechanism of ketamine action implicate the conversion of ketamine into an abundant distinct metabolite known as hydroxynorketamine (HNK) found both in human and rodent plasma. HNK seems to be critical in the increase in presynaptic glutamate release, and inducing early and sustained activation of the α-amino-3-hydroxy-5-methyl-4-isoxazole-propionic acid receptor (AMPAR) relative to the NMDAR in hippocampus (32, 33). In fact, Zanos et al., in a series of well-controlled experiments, demonstrated that HNK exerted antidepressant effects on forced swim test and learned helplessness tests independent of NMDAR effects, a process that appears to be related to physiologic activation of AMPAR in the hippocampus and medial prefrontal cortex (32). Blockade of AMPA receptors in these loci with the specific AMPA blocker NBQX abolished the antidepressant effects of ketamine (32). Furthermore, Zanos et al., found that at relevant antidepressant concentrations (10 µM) (2R,6R)-HNK neither inhibited NMDARs nor induced any of the side effects typically associated with ketamine (32, 34). However, at this stage, there are no human studies to substantiate the antidepressant properties of HNK in humans.

Intriguingly, recent work from Yang et al. proposed that inhibition of lateral habenula glutamatergic neurons may be an additional NMDA-dependent mechanism. Their work revealed that local administration of ketamine or other compounds that block NMDAR-inhibited bursting activity of the lateral habenula had a rapid antidepressant effect (35). This group also found that photo-stimulation of lateral habenula drives behavioral despair and anhedonia, which is blocked by inhibiting the NMDA bursting activity effects on monoaminergic reward centers (35). While the blockade of NMDA receptors in the lateral habenula was sufficient to reverse experimentally induced behavioral despair, blockade of low-voltage-sensitive T-type voltage sensitive calcium channels (T-VSCC) was also sufficient to induce rapid-antidepressant effects (35). This work provides a simpler model whereby ketamine quickly (minutes to hours) elevates mood by blocking NMDAR-dependent burst activity of lateral habenula neurons and, in turn, disinhibits downstream monoaminergic reward centers (35). However, it remains unclear whether NMDAR-dependent antagonism alone may be sufficient for ketamine’s fast onset and protracted antidepressant effects. While this hypothesis needs to be substantiated in clinical studies, non-NMDAR-mediated glutamatergic potentiation and sustained activation of AMPARs seem to be central to the long-lasting antidepressant effect of ketamine and its main metabolite, HNK.

In an animal model of depression, the learned helplessness test, the lateral habenula activity is significantly increased, and the activity of brainstem dopaminergic neurons was concomitantly inhibited (19). The increase in lateral habenular firing rate resolved with the administration of antidepressants (19). In a congenital animal model of learned helplessness, the lateral habenular firing rate was also increased, which resolved after antidepressant administration (36). In models of depression provoked by maternal separation, lateral habenular firing was increased, but resolved with interventions that normalized the lateral habenular firing rate (37).

Further, a complete pharmacologic stereotaxic inhibition of lateral habenular firing bursts ameliorated depression-like behaviors in rodents and ameliorated the decreased raphe nucleus firing and serotonergic neurotransmission associated with increased habenular neuronal activity (36). The latter finding is compatible with work showing that activation of the lateral habenula inhibits the serotonergic raphe nuclear firing rates. In addition, Yang et al. show that acute ketamine treatment inhibits NMDAR-dependent burst activity in the lateral habenula, resulting in the disinhibition of the downstream activity of midbrain dopaminergic neurons and serotoninergic neurons, which are responsible for activating the reward centers in the brain. As noted, local blockade of NMDARs or low-voltage-sensitive T-type voltage sensitive calcium channels (T-VSCCs) in the lateral habenula sufficed to induce rapid antidepressant effects (35). Furthermore, in an animal model of maternal-deprivation-induced severe early life stress, a single *in vivo* administration of ketamine induced long-lasting antidepressant effects as well as the reversal of lateral habenula neuronal dysfunction up to 72 h post-injection (38).

Prior research has shown that lateral habenula and the serotoninergic neurotransmission are bidirectionally interconnected, but the functional role of this interconnection has been largely elusive. Recently, scientists have been able to shed light into this functional interconnectivity using optogenetic or pharmacological approaches through perturbation of serotonin signaling, which influences lateral habenula activity. Indeed, tryptophan depletion in patients with depressive illness increases cerebral blood flow in the lateral habenula and initiates increased firing rates, indicated by the lateral habenula’s capacity to down-regulate raphe nucleus activity in patients with depressed mood (24). Moreover, Carlson et al. showed that single-dose ketamine infusion in treatment-resistant major depressive disorder (MDD) patients abolishes glucose hypermetabolism in lateral habenula compared to baseline (39), indicative of normalization of habenular brain activity following ketamine infusion.

A postmortem histologic study in MDD patients showed not only decreased size of the lateral habenula but also a reduction in the total number of lateral habenular neurons (40). While limited in number and power, the habenular volumetric alteration studies have produced mixed results for the field so far, in part due to limited spatial resolution and delineation of the structure. For example, a previous study in patients with depressive illness revealed volumetric reduction of the lateral habenula that was most prominent in bipolar depressed patients and in female patients undergoing a current major depressive episode (41). In contrast, Liu et al. show increased habenula volume in unmedicated depressed subjects compared to healthy volunteers (42). Interestingly, the study showed a positive association of habenula volume and more severe anhedonia scores. Consistent with that, other studies have found that greater habenula volume was associated with onset of first-episode depression (43) and correlated with depression symptom severity scores (44).

Deep brain stimulation of the lateral habenula of a patient with severe treatment-resistant depression completely abolished depressive symptomatology. Intriguingly, an inadvertent cessation of stimulation, the subject experienced a relapse of depression but regained remission after the reinstatement of habenular stimulation (22).

Lawson et al. found that, in healthy volunteers, lateral habenula activation increased as conditioned stimuli became more strongly associated with electrically induced shocks. This pattern was significantly different in depressed subjects, for whom habenula activation decreased significantly with increasing association between conditioned stimuli and electric shocks. In both volunteers and patients, individual differences in habenula volume were negatively associated with symptoms of anhedonia (20). In this study, depressed subjects exhibited abnormal negative task-related habenula responses during aversive conditioning. The direction of this effect is opposite to that predicted by other accounts of depression based on findings in animal models. The authors speculate that the negative habenula responses may result by the loss of the capacity to actively avoid negative cues in MDD, which could lead to excessive negative focus (8, 20).

#### Molecular Mechanisms

While lateral habenula neurons are primarily glutamatergic, its input come from discrete brain areas and are both excitatory (glutamatergic) and inhibitory (GABAergic) in nature (3). These inputs are integrated into complex bidirectional downstream regulatory fashion with the monoaminergic system *via* both direct and indirect connections to the VTA. In addition, the lateral habenula indirectly inhibits dopaminergic neurons in VTA and serotoninergic neurons in raphe nuclei through GABAergic RMTg, conveying information indicative of negative reward and aversive stimuli (15, 45). In animal models, lateral habenular circuits are involved in behavioral avoidance and inhibition of motor response appraised through various afferent circuits and efferent circuit to RMTg (45). Experimental activation of lateral habenular circuits in animal models produces active, passive, and conditioned behavioral avoidance (2). As noted, direct inhibition of lateral habenular NMDA receptors either optogenetically or *via* the use of ketamine produces rapid-acting antidepressant effects in multiple animal models of depression-like syndromes (35).

Cui et al. demonstrated that an astroglial potassium channel Kir4.1 is up-regulated in the lateral habenular model of depression, associated with an increased firing rate of the lateral habenula (46). Loss of Kir4.1 in the lateral habenula ameliorates the increased firing rate of this structure and resolves depressive symptomatology (46). Specific gain of Kir4.1 in the lateral habenula, on the other hand, promotes depressive symptomatology (46). Thus, Kir4.1 in the lateral habenula may serve as a conceivable target for the drug treatment discovery in depression.

The mechanism by which an up-regulated Kir4.1 mediates depression has not been definitively established. Recent data indicate that up-regulated Kir4.1 may lead to neuronal hyperpolarization, inactivating T-type voltage-sensitive calcium channels, which in turn lead to NMDAR bursts that ultimately result in increased suppression of downstream monoaminergic centers. Hence, as noted, ketamine blockage of lateral habenula NMDA receptors results in amelioration of depression-like symptoms in rodents (35).

P11 is another multifunctional protein that interacts with serotonin receptor enzymes, chromatin remodeling factors, and ion channels, which are critically involved in depression-like behaviors and antidepressant actions (18). p11 is enriched in distinct neuronal types, especially in the nucleus accumbens. A previous study revealed that chronic stress leads to an increase in p11 in dopamine D2 neurons, which contributes to behaviors suggestive of depression in an animal model of depression-like behaviors in animal models. P11 is also significantly increased in the lateral habenula of chronicly stressed animal models (18). Specific knockout of p11 in the lateral habenula alleviates the stress-induced depressive behaviors. On the other hand, overexpression of p11 in the lateral habenula results in depressive behaviors (18). Thus, p11 may appears to have a key role in the pathophysiology of depression and an interesting target for pharmacological intervention.

Using a quantitative proteomic screen, Li et al. discovered a signaling pathway enzyme known as the β form of calcium/calmodulin-dependent protein kinase type II (βCaMΚΙΙ), which, when overexpressed in the lateral habenula, produced depressive-like behaviors (47). Manipulations increasing the presence of βCaMΚΙΙ consistently increased depressive behaviors, including anhedonia and behavioral despair (48). In contrast, antidepressant medications and RNA interference of βCaMΚΙΙ significantly diminished depressive manifestations (48). The mechanisms by which up-regulation of βCaMΚΙΙ leads to increased firing rates of the lateral habenula have not been definitively determined, yet βCaMΚΙΙ, nevertheless, represents a potential target for ameliorating depressive symptomatology.

Stressful stimuli that increase lateral habenular firing rates and promote a depressive behavioral phenotype increase protein phosphatase 2A (PP2A), which has known influence on the functional activity of the GABA_B_ receptor that regulate G-coupled inwardly rectifying potassium channel receptor (GIRK) (49). Specifically, chronic stress causes significant weakening of GABA_B_ GIRK function and neuronal excitability, which is restored by pharmacologic inhibition of PP2A (49). Thus, PP2A inhibitors may have therapeutic efficacy in depressive syndromes associated with increased firing of lateral habenula neurons. More so, recent data indicate that pharmacological activation GABA_B_ receptor or axon-sparing lesion in the RMTg, a region that receives dense projection from lateral habenula, significantly suppresses the lateral habenula firing rate (50). Hence, the restoration GABA_B_ signaling may ameliorate depression symptomatology (49).

In sum, the confluence effect of stress-induced up-regulation of Kir4.1, p11, βCaMKII, and PP2A activity in lateral habenula contributes to amplification of its firing bursts activity and subsequent inhibition of monoaminergic reward centers, therefore contributing to depressive symptomatology. This research suggests that parallel cellular processes converge in the lateral habenula to transduce the impact of aversive stimuli.

## Key Concepts

1. Aversive and invalidating environmental stimuli individually or coupled with chronic stress precipitate increased firing of the lateral habenula, leading to down-regulation of brainstem dopaminergic neurotransmission and decreased activity of the nucleus accumbens, inducing anhedonia and depressive symptomatology.
2. Increased lateral habenular firing also leads to down-regulation of the serotonergic neurons in raphe nuclei neurons and decreased serotonergic neurotransmission, known to be implicated in depressive symptomatology.
3. In contrast, outcomes that meet or exceed expectations lead to decreased lateral habenular firing and increases in dopaminergic and serotonergic neurotransmission. The ability to update expectations of the environment based on rewards and disappointments, reflects a potentially important survival mechanism relevant to the capacity to adapt to ever-changing environmental circumstances.
4. Multiple lines of evidence derived by both preclinical and clinical studies confirm the relationship between stimuli that activate the firing rate of the lateral habenula and the appearance of depressive symptomatology. Postmortem studies clinical finding of a smaller lateral habenula in depressed patients, while human clinical imaging studies show increased glucose utilization of this structure, which normalizes following ketamine treatment.
5. Broadly, ketamine-induced rapid antidepressant and antianhedonic effects are believed to be mediated by a) direct inhibition of spontaneous synaptic NMDAR in the prefrontal cortex and hippocampus or inhibition of NMDAR-burst activity in lateral habenula, b) indirect inhibition of NMDAR at a presynaptic GABAergic interneuron site, or c) conversion of ketamine to its active metabolite HNK, inducing an early and sustained activation of AMPAR at hippocampus, proving for the first time that NMDAR inhibition is not essential for the antidepressant effects of ketamine.
6. Direct injection of ketamine into the lateral habenula, an anti-reward center abundant in NMDA receptors, induces rapid amelioration of experimentally induced depressive-like syndromes in animal models.
7. Aversive stimuli induce up-regulation of the Kir4.1 or p11 is associated with rapid firing rates in the lateral habenula and induces depression-like phenotype, while pharmacological down-regulation or knockout manipulation in animal models is associated with resolution of depression-like symptoms.
8. Increasing the presence of the enzyme βCaMKII consistently increased depressive behaviors, including anhedonia and behavioral despair. In contrast, antidepressant medications and RNA interference of βCaMKII significantly diminished depressive manifestations.
9. Chronic stress causes significant weakening of GABA**_B_**–GIRK pathway function and neuronal excitability in lateral habenula, which is restored by pharmacologic inhibition of PP2A. Thus, PP2A inhibitors may have therapeutic efficacy in depressive syndromes associated with increased firing of lateral habenula neurons.
10. The HPA axis and the lateral habenula appear to be intricately interconnected to regulate the stress-related adaptive response to aversive stimuli.
11. Increase in lateral habenula firing rate induces decrease in slow wave activity through the influence of serotonin neurons in the VTA and raphe nuclei, whereas the augmentation of REM sleep activity appears to be mediated by descending brainstem circuits *via* RMTg.
12. Strategies aimed at decreasing the firing rate of the lateral habenula show great promise for significant amelioration of associated depressive symptomatology.

## Author Contributions

PG and BK designed the contents of this article, interpreted the data, and wrote the review. Both authors read and approved the final manuscript.

## Funding

This work was supported by the Intramural Research Program at the National Institute of Mental Health, National Institutes of Health (IRP-NIMH-NIH).

## Conflict of Interest Statement

The authors declare that the research was conducted in the absence of any commercial or financial relationships that could be construed as a potential conflict of interest.
